# Acute copper deficiency myelopathy after single-anastomosis gastric bypass

**DOI:** 10.1093/omcr/omad138

**Published:** 2023-12-19

**Authors:** Georgia Taylor, Eshwarshanker Jeyarajan

**Affiliations:** Cairns Base Hospital, General Surgery, Cairns North, QLD, Australia; Cairns Base Hospital, General Surgery, Cairns North, QLD, Australia

**Keywords:** copper deficiency, myelopathy, bariatric surgery

## Abstract

Bariatric surgery is a well-established treatment for morbid obesity, combining both restrictive and malabsorptive mechanisms to achieve weight loss. Macro and micronutrient deficiencies are some of the most common complications of these operations, which in rare occasions can be unexpected, severe, and difficult to manage. We present a case of severe copper deficiency related myelopathy in a patient post single anastomosis gastric bypass, requiring parenteral copper replacement and eventual reversal. She presented with ascending lower limb paraesthesia and weakness, with copper levels on admission of 4 μmol/l, and ceruloplasmin 94 mg/l. She continued to have progressive neuropathy and visual deterioration, despite IV and enteral replacement, and eventually underwent reversal of her bypass, with normalization in her copper levels and incomplete improvement in symptoms. Copper deficiency myelopathy is a rare and severe complication of bariatric surgery. Early identification is key, as neurological symptoms are often not reversible.

## INTRODUCTION

Bariatric surgery is a well-established and effective treatment for morbid obesity, combining both restrictive and malabsorptive mechanisms to achieve radical and sustained weight loss. Macro and micronutrient deficiencies are some of the most common complications of these operations, which in rare occasions can be unexpected, severe, and difficult to manage.

The most common deficiencies are iron, folic acid, vitamin D and B12; less common are other micro-elements such as copper, zinc and selenium [1]. Diagnosis and management of these deficiencies remains a key element in follow up for bariatric surgery patients.

Myelopathy associated with copper deficiency is a relatively new entity, first being described in 2001 [2]. It presents very similar to B12 deficiency, with sensory ataxia, gait disturbances, and paraesthesia in extremities [3]. It has been associated with various upper gastrointestinal surgeries, including partial gastrectomy and roux-en-Y gastric bypass (RYGB). Treatment is with oral and IV copper replacement, however most patients do not gain total resolution of their neurological symptoms [3]. Here we present a case of severe copper deficiency myelopathy caused by the combination of single anastomosis gastric bypass (SAGB), zinc supplementation and gastroenteritis.

## CASE PRESENTATION

A 44-year-old female presented to the Emergency Department with a 4-week history of ascending lower limb weakness and paraesthesia beginning one week after an episode of campylobacter gastroenteritis with poor oral intake and diarrhoea, while concurrently being treated with oral zinc replacement for zinc deficiency. Her medical history was significant for a SAGB two years previously, completed to facilitate weight loss prior to a hysterectomy for endometriosis, she had lost 68 kg.

On examination, power was 3/5 in proximal lower limb groups, and 2/5 in distal groups, upper limbs were less affected with 5/5 power in shoulders and 4/5 in all other groups. She was noted to be hyperreflexic in her knee and ankle jerk reflexes, with upgoing plantars, and had stocking distribution sensation loss for pain (to T11), and temperature and vibration (below knees).

She has a mild normocytic anaemia (Hb 104) otherwise routine bloods were unremarkable. Initial differentials included Guillian-Barré and transverse myelitis. CT brain and spine were unremarkable, as was a lumbar puncture. An MRI showed multifocal areas of high T2 signal/cord oedema at C3-6 and T2 in keeping with cord myelopathy, consistent with a demyelinating process. Copper and ceruloplasmin levels were completed and were 4 μmol/l (normal 11–24 μmol/l) and 94 mg/l (normal 200–390 mg/l) respectively. She was diagnosed with copper deficiency myelopathy.

She was commenced on oral and IV copper replacement, however, was unable to substantially increase her copper levels despite multiple attempts at IV replacement (copper 6 μmol/l, ceruloplasmin 158 mg/l) over a 5 month period and had progressive neurological symptoms leaving her unable to walk.

She underwent a diagnostic laparoscopy with on table upper gastrointestinal endoscopy, which showed a SAGB, and an unusually long small bowel of nine metres; six metress was biliopancreatic limb and three metres of common channel. Placement of feeding gastrostomy was completed at this time. Unfortunately, the patient was unable to tolerate feeding with the gastrostomy, and despite continued IV replacement her copper levels remained severely low (copper 6 μmol/l, ceruloplasmin 84 mg/l) she then consented to reversal of the SAGB. She underwent the procedure 9 months after the onset of her symptoms and had rapid return of her copper levels (8 days post copper was 20 μmol/l, ceruloplasmin 308 mg/l). She had significant, although not complete, resolution of her neurological symptoms, with improvements in strength and paraesthesia, however, still remains wheelchair dependent for mobility.

## DISCUSSION

Copper is an essential dietary micronutrient with significant roles in key enzymes affecting the neurological, haematological and immunological systems. While best known for its accumulation in Wilson’s disease, the vast growth in bariatric surgery is making copper deficiency a more common entity [3].

Copper is absorbed predominantly from the proximal duodenum and stomach. In the blood it binds albumin and is transported via the portal system to the liver. Within the liver it binds ceruloplasmin for storage. Excretion is via biliary loss, however saliva, sweat and GI secretions also contribute [4]. Guidelines currently recommend a copper intake of 1.2–1.7 mg/day to remain replete [5].

Micro- and macro-nutrient deficiencies are a common complication post bariatric surgery. Reports show a prevalence of copper deficiency post RYGB of 10–12% in the first three years post-operatively [1], with incidences reported up to 18.8% [6]. With routine supplementation, rates range from 0–5% [7].

The majority of patient with copper deficiency are either asymptomatic or experience mild symptoms. The most common presentation is with anaemia which may be micro-, normo-, or macrocytic; leucopoenia and thrombocytopaenia are also possible [4].

Current guidelines recommend routine supplementation in the form of a multivitamin containing copper for all bariatric patients, with testing of levels for any patients with anaemia, neutropoenia, myeloneuropathy, or impaired wound healing [8].

Zinc excess or supplementation had regularly been described as a cause of severe copper deficiency. Often reported from overuse of denture adhesive cream, however more recently iatrogenic causes, such as gastrointestinal or bariatric surgery, are responsible. Zinc upregulates the heavy metal chelator metallothionein in enterocytes, for which copper has a higher affinity than zinc. In our case, the oral zinc replacement caused copper wastage and precipitated our patients initial neurological decline [3].

Limb length measurements in bariatric surgery remains controversial. We hypothesize that our patient had her common channel length measured backwards from the ilocaecal valve, resulting in a large proportional bypassing, contributing to her deficiency. An RCT has shown greater weight loss is achieved with longer biliopancreatic limbs in RYGBs, however also found greater rates of nutritional deficiencies in this group [9].

Copper deficiency is an uncommon complication of bariatric surgery and can be severe and life-threatening. Consider routine supplementation with copper containing multivitamin for all bypass patients and maintain a low threshold for copper testing in any patients with anaemia or progressive neuropathy. Early identification of copper deficiency myelopathy is key, as neurological symptoms are often not reversible [3].
